# Super-Enhancer-Associated Gene 3-Hydroxybutyrate Dehydrogenase 1 Increases Intramuscular Fat Deposition of Yaks (*Bos grunniens*)

**DOI:** 10.3390/foods15101811

**Published:** 2026-05-20

**Authors:** Xue Meng, Jieqiong Ma, Yanjie Yin, Zhenlu Xie, Binglin Yue, Hui Wang

**Affiliations:** 1 Key Laboratory of Qinghai-Tibetan Plateau Animal Genetic Resource Reservation and Utilization, Sichuan Province and Ministry of Education, Southwest Minzu University, Chengdu 610041, China; mx20170924@163.com (X.M.); 17381570059@163.com (J.M.); 15351407229@163.com (Y.Y.); zhenluxie0606@163.com (Z.X.); yuebinglin123@163.com (B.Y.); 2 Key Laboratory of Animal Science of State Ethnic Affairs Commission, Southwest Minzu University, Chengdu 610041, China

**Keywords:** yaks (*Bos grunniens*), intramuscular fat content, super-enhancers, *BDH1*

## Abstract

Intramuscular fat (IMF) is a pivotal determinant of meat quality in yaks (*Bos grunniens*). While nutritional factors are well-documented, the epigenetic landscape, particularly the transcriptional architecture governed by super-enhancers (SEs), remains largely unexplored in the context of IMF deposition. To investigate SE-associated genes, Chromatin immunoprecipitation sequencing (ChIP-seq) assays using H3K27ac antibodies and RNA-sequencing (RNA-Seq) were conducted on *longissimus dorsi* (LD) muscle tissues with high and low IMF contents. Integrated multi-omics analysis identified 82 enhancer-associated genes exhibiting significant upregulation in high-IMF samples, with 63 loci characterized as SE-associated. In particular, H3K27ac signal distribution analysis indicated that SEs were distributed across functional regions such as promoters, gene bodies, exons, and introns. Among these SE-related genes, 3-hydroxybutyrate dehydrogenase 1 (*BDH1*) was further investigated to understand its function and regulatory mechanisms. To address this, overexpression or knockdown experiments were conducted, followed by CCK-8, EdU, Bodipy functional assays, and Real-time quantitative PCR (RT-qPCR) analysis. Functional experiments revealed that *BDH1* acts as a key positive regulator of yak preadipocyte differentiation and is a prime SE-associated candidate regulatory gene. Furthermore, dual-luciferase reporter assays were performed to identify its SE region, revealing that the activity of 4 enhancer regions was significantly upregulated. Collectively, these findings implicate SE-associated genes in IMF deposition in yaks, provide a valuable resource for future research, and underscore the functional relevance of *BDH1* in this process.

## 1. Introduction

As a native ruminant uniquely adapted to the Qinghai–Tibet Plateau, the yak (*Bos grunniens*) represents a cornerstone of China’s high-altitude genetic resources [1]. Although it is famous for lean, nutrient-dense meat, its naturally low intramuscular fat (IMF) content reduces the tenderness and flavor, thereby limiting the improvement of its commercial value [2]. Compared to ordinary cattle of the same age, yaks display slower growth rates, poor muscle development, reduced fat deposition efficiency, and lower tenderness, hindering the economic value of yak meat [3]. IMF content is significantly correlated with indicators such as meat color, tenderness, juiciness, and flavor; hence, determining the quality of meat [4]. IMF refers to the fat stored within skeletal muscles, which are composed of lipid droplets within muscle cells [5]. IMF deposition is a complex biological process, influenced by nutritional factors including energy, proteins, and trace elements [6,7,8]. It is also affected by vital genes that regulate IMF deposition in livestock. A transcriptome analysis was conducted on the Small-tailed Han sheep and their Suffolk crossbreds. The results indicated that a peroxisome proliferator-activated receptor α may be a key gene regulating IMF deposition [9]. Integration and analysis of transcriptome and metabolome data of Beijing Black pigs revealed that *ADIPOQ*, *C1QDC*, and *DGAT2* are related to IMF deposition, and *KLHL40* is a novel regulator in this process [10]. Transcriptional profiles in subcutaneous and intramuscular adipose of Japanese Black cattle demonstrated that *CPE* and *COL4A5* are preferentially expressed in IMF tissue, and indicated the involvement of TGF-β pathway-related regulatory factors [11]. Numerous studies have elucidated the genetic regulatory mechanisms underlying IMF deposition in pigs, sheep, and ordinary cattle, and identified critical candidate genes, regulatory factors, and signaling pathways. However, research on the genetic underpinnings of IMF deposition in yaks is limited.

During mammalian development and differentiation, enhancers, as modular DNA elements, play a crucial role in determining cell identity. These cis-regulatory sequences consist of several hundred base pairs (bp) and coordinate the spatiotemporal expression of target genes by serving as docking platforms for transcription factor arrays [12,13,14,15]. Super-enhancers (SEs) are dense clusters of enhancer elements, rich in abnormally high concentrations of transcriptional co-activators and machinery [7]. This unique composition enables them to exert dominant control over the gene expression programs that define cellular identity. This unique structure makes it possible to achieve concentrated activation of genes related to cell identity, and has great potential in elucidating the mechanistic basis of developmental processes and pathological conditions, including tumorigenesis [16,17,18]. However, the role of SE-associated genes in IMF deposition in yaks largely remains unexplored.

To identify differentially expressed genes (DEGs) regulated by SEs, ChIP-seq and RNA-Seq combined analysis was conducted on the LD tissues of yaks with high and low fat content, and overexpression and interference techniques were used to investigate the effect of SE-related genes on the proliferation and differentiation of preadipocytes. This study provides fundamental data for elucidating the regulatory roles of SE-related genes in IMF deposition and offers important reference information for yak genetic breeding.

## 2. Materials and Methods

### 2.1. Sample Collection and IMF Content Determination

Twenty-four male Maiwa yaks of comparable body weight were reared under natural grazing conditions in the alpine pastures of Xiaojin City, Sichuan Province, under the same grazing management until the age of four. After that, these animals were fattened for a period of six months on the same diet. Following humane euthanasia, LD muscle samples were collected [19]. IMF content was measured using the Soxhlet extraction technique [20]. For high-throughput sequencing analysis, six representative individuals were selected and divided into two groups: Three individuals exhibited higher IMF concentration (4.38 ± 0.01%), and the other three demonstrated a lower level (2.71 ± 0.19%). An independent (unpaired) *t*-test was used to assess the statistical significance of IMF content determination between the H-IMF and L-IMF groups. The difference was statistically significant (* *p* < 0.05).

### 2.2. Cell Culture and Treatment

LD muscle samples were harvested from the 12th to 13th rib region of yaks; the tissues were washed with phosphate-buffered saline (PBS) containing 3% antibiotic mixture (penicillin/streptomycin). Tissue samples were homogenized in digestion buffer and subsequently digested with 2.5 mg/mL Type II Collagenase (Sigma-Aldrich, St. Louis, MO, USA) for 3 h at 37 °C under continuous gentle agitation. The cell suspension was washed with HBSS (Thermo Fisher Scientific), filtered through 70 µm and 40 µm nylon filters, and centrifuged (2000× *g* for 5 min). The pellet was resuspended in erythrocyte lysis buffer, centrifuged, and washed twice with Dulbecco’s Modified Eagle Medium (DMEM) (Thermo Fisher Scientific, Waltham, MA, USA). Differential centrifugation (1800 and 1600 rpm, 5 min each) was performed to enrich the cell population [21]. The purified cell fraction was resuspended in a growth medium prepared with DMEM/F12 (89%), fetal bovine serum (10%), and penicillin/streptomycin (1%). Cells were maintained at 37 °C in a humidified atmosphere of 5% CO_2_ using a Thermo Scientific Forma 3111 incubator. The growth medium was replaced after an initial adherence period of 1.5 h [21,22].

### 2.3. RNA-Seq Analysis

Total RNA was extracted with TRIzol kits (Invitrogen, Carlsbad, CA, USA) according to the manufacturer’s instructions. Poly (A)+ mRNA was isolated from total RNA using Oligo(dT) magnetic beads. The enriched mRNA was fragmented into short pieces of ~200 nt. First-strand cDNA was synthesized using random hexamer primers and fragmented RNA as a template, followed by second-strand cDNA synthesis. The resulting double-stranded cDNA underwent end-repair, A-tailing, and ligation with specific sequencing adapters. PCR was performed to enrich the adapter-ligated cDNA fragments and generate the sequencing library. Library quality and quantity were assessed using an Agilent 2100 Bioanalyzer (Agilent Technologies, Santa Clara, CA, USA) and the ABI StepOnePlus Real-Time PCR System (Thermo Scientific, Waltham, MA, USA). Sequencing was conducted on the Illumina HiSeq platform (Illumina, Inc., San Diego, CA, USA).

### 2.4. ChiP-Seq Experiment

ChIP assays were performed following standardized methodologies [23]. Briefly, ~3 g of lumbar skeletal muscle tissue was homogenized and subjected to cross-linking with 1% formaldehyde at 20 °C for 20 min. Cross-linking was terminated by introducing glycine (2 M), followed by a 5 min incubation under identical thermal conditions. Following fixation, chromatin was fragmented to a size range of 200–500 bp via focused ultrasonication using a Covaris M220 instrument (Covaris, Woburn, MA, USA). For ChIP, acetylated histone complexes were selectively enriched with 3 μg of anti-H3K27ac antibody (ab4728, Abcam, Cambridge, UK), enabling specific pull-down of target chromatin regions. To rigorously assess non-specific background binding, parallel control reactions were performed using isotype-matched rabbit IgG (ab172730, Abcam, Cambridge, UK). Reversal of formaldehyde cross-links was achieved via overnight incubation at 65 °C with proteinase K. The liberated DNA fragments were purified through the MinElute PCR Purification Kit (Qiagen, Hilden, Germany). Quality assessment of the constructed libraries was performed through microfluidic electrophoresis and fluorometric quantification, utilizing instrumentation based on Agilent’s 2100 Bioanalyzer (Santa Clara, CA, USA) and Thermo Fisher’s Qubit 3.0 systems (Waltham, MA, USA), respectively, to ensure integrity and concentration accuracy before paired-end sequencing on a next-generation sequencing platform compatible with Illumina chemistry.

### 2.5. ChiP-Seq Data Analysis

Raw sequencing reads were processed to trim adapter sequences and filter out low-quality reads using the fastp software (version 0.19.11), thereby generating high-fidelity clean data suitable for downstream bioinformatics analysis. The high-quality reads were mapped to the reference genome of the yak (BosGru, version 2.0) employing the Burrows–Wheeler Aligner (BWA, version 0.7.12.r1039). Following alignment, peak calling was executed using MACS2 (version 2.1.0) with default settings and a false discovery rate cutoff of 0.05. To characterize the genomic landscape of the identified peaks, their features were systematically evaluated via the Cis-regulatory Element Annotation System, covering parameters ranging from chromosomal distribution and peak width to enrichment fold, statistical significance, and summit location. Concurrently, de novo motif enrichment was interrogated via the Homer suite (version 4.9.1) using the findMotifsGenome.pl script. Differential peak analysis was executed using the DiffBind package (version 1.16.3), with significantly differential peaks defined by a fold-change threshold exceeding 2. The ChIPseeker R package (version 1.30.3) was leveraged to determine the genomic distribution of peak summits relative to key functional gene elements, specifically 5′ untranslated regions (UTRs), protein-coding sequence (CDS), and 3′ -UTRs. ChIP-seq signal profiles, including heatmaps and average enrichment tracks, were visualized using DeepTools (version 2.5.4), facilitating the interpretation of regional chromatin activity. SEs were subsequently delineated through the Rank Ordering of Super-Enhancers algorithm to identify regulatory elements with exceptionally high mediator occupancy [24]. A gene was considered associated with an SE if its transcription start site or hairpin sequence was located within the SE region, or if its association score was below 0.2 [25].

### 2.6. Combine Analysis of ChiP-Seq and RNA-Seq Data

To interrogate the functional link between histone modification and gene expression, H3K27ac ChIP data were correlated with transcriptomic profiles. This integrative approach was employed to evaluate the association between enhancer/promoter occupancy (H3K27ac) and transcriptional output. Genomic regions identified as peaks in ChIP-seq analysis were intersected with DEGs derived from RNA-seq, enabling the identification of genes under significant regulatory influence of H3K27ac modifications. Quantification of ChIP-seq signal intensity was standardized using reads per kilobase per million mapped reads normalization. Concurrently, transcript abundance measurements were normalized to transcripts per million to ensure cross-sample comparability of gene expression levels. To interrogate the biological implications of significantly regulated genes, annotation resources from gene ontology (GO) (http://www.geneontology.org/) and KEGG (http://www.genome.jp/kegg/, accessed on 16 May 2022 in Chengdu, Sichuan, China) pathways were systematically deployed. Computational enrichment analysis was executed through ClusterProfiler (version 3.8.1), ensuring statistical rigor with a significance cutoff of * *p* ≤ 0.05 for enriched biological processes and molecular pathways [26].

### 2.7. Systematic Enrichment Profiling of Biological Functions

To elucidate the primary biological roles, functional enrichment analyses were conducted on the source gene set. To assign biological functions, all identified genes were cross-referenced with the authoritative GO (http://www.geneontology.org/) and Kyoto encyclopedia of genes and genomes (KEGG) (http://www.kegg.jp/kegg/, accessed on 20 July 2022 in Chengdu, Sichuan, China) repositories. Statistical significance was stringently evaluated to identify enriched terms. Only GO categories and KEGG pathways exhibiting a corrected *p* < 0.05 were retained as significant.

### 2.8. Cell Transfection

Yak intramuscular preadipocytes (YIMAs) were cultured under a humidified atmosphere of 5% CO_2_ at 37 °C. The growth medium consisted of DMEM supplemented with 10% fetal bovine serum (FBS; Gibco, Waltham, MA, USA) and 1% penicillin-streptomycin (Boster Bio, Pleasanton, CA, USA). Upon reaching 80% confluence, cells were subjected to genetic intervention. Specifically, Lipofectamine 3000 (Invitrogen, Carlsbad, CA, USA) was employed to perform transient transfection, introducing either the 3-hydroxybutyrate dehydrogenase 1 (*BDH1*) overexpression construct or specific siRNA inhibitors, in conjunction with their respective negative control. Following a 6 h incubation period to allow for nucleic acid delivery, the transfection mixture was replaced with a specialized differentiation medium. This induction medium was formulated by supplementing the standard growth medium with 50 μM oleic acid (Sigma-Aldrich, St. Louis, MO, USA), and cells were subsequently cultured in this environment for a duration of 48 h to facilitate differentiation.

### 2.9. RNA Extraction, Reverse Transcription, and Real-Time Quantitative PCR

Total RNA was extracted from adipocytes with TRIzol reagent (Takara, Shiga, Japan), followed by reverse transcription using the PrimeScript RT Master Mix (Takara, Dalian, China). Gene expression levels were quantified by RT-qPCR using the SYBR Premix Ex Taq kit (Takara, Dalian, China) on the LightCycler 96 System (Roche, Munich, Germany). GAPDH was used as an internal control to normalize the expression data. To ensure data reliability, the experimental design incorporated triplicate biological samples alongside three technical replicates for each assay. Relative gene expression was normalized to internal controls using the comparative 2^−ΔΔCt^ algorithm. Gene-specific primers were synthesized by Tsingke Biotech (Beijing, China) and are presented in detail in Table S1.

### 2.10. Oil Red O and Bodipy Staining

Adherent cells cultured in 12-well plates were harvested and fixed with 10% formaldehyde at 20 °C for 30 min. Following quenching of the fixative with two PBS washes, cells were subjected to a dual-color staining protocol. This involved a 1 h incubation with a co-staining solution containing Oil Red O (Sigma) and Bodipy (Invitrogen). Notably, the Oil Red O working solution was formulated by blending PBS with the isopropanol stock solution in a 2:3 proportion. After stringent washing to remove unbound fluorophores, stained lipid droplets were examined and imaged using bright-field microscopy.

### 2.11. Cell Proliferation Assay

To evaluate cellular expansion, a dual-assay approach was employed using the CCK-8 (Vazyme, Nanjing, China) and EdU (Beyotime, Shanghai, China) detection systems, strictly adhering to the provided technical specifications. Briefly, cells were seeded in 96-well plates, targeting a final concentration of 1 × 10^3^ cells per well. Upon achieving 70–80% confluence, the cultured cells were subjected to genetic manipulation via transfection with either *BDH1* overexpression constructs, empty vector controls, or si*BDH1* oligonucleotides. Post-transfection, cell viability was monitored at 12, 24, 48, and 72 h. Specifically, CCK-8 reagent (10 μL) was introduced into each well, and samples were incubated at 37 °C in 5% CO_2_ for 1 h before absorbance measurement at 450 nm on a microplate reader. For the EdU assay, at 48 h post-transfection, EdU was introduced into the culture medium for a 2 h pulse to label proliferating cells, with all incubations conducted at 37 °C in 5% CO_2_ to ensure stable experimental conditions. Cell fixation was performed using 4% paraformaldehyde for 30 min to preserve structural integrity, followed by EdU detection procedures as guided by the manufacturer’s protocol for the EdU-594 labeling kit. Fluorescence microscopy was employed to visualize and capture images of EdU-positive cells.

### 2.12. Dual-Luciferase Reporter Gene Assay

To evaluate the regulatory potential of the putative enhancers (E1–E5) against an NC, specific DNA fragments were amplified via PCR (primers listed in Table S2) and subcloned into the pGL3-Promoter vector (Promega, Madison, WI, USA). This subcloning strategy employed *Mlu* I and *Bgl* II restriction enzymes (NEB, Ipswich, MA, USA) to facilitate directional ligation. The resulting recombinant constructs were introduced into YIMAs cells cultured in 12-well plates via transient transfection. To eliminate the differences in transfection efficiency, we transfected the cells with the pRL-TK plasmid simultaneously as a standardized reference. 48 h after transfection, the dual luciferase reporter gene detection system (Promega, E1910) was used to assess the activity of the reporter gene. Firefly luciferase signals were adjusted relative to Renilla luciferase levels in each sample. All experiments were conducted in triplicate to ensure reproducibility.

### 2.13. Statistical Analysis

Data are expressed as mean ± standard error of the mean (SEM). For the sequencing data, an independent (unpaired) *t*-test was used to assess the statistical significance of IMF content between the H-IMF and L-IMF groups. In the subsequent cell experiments, a paired *t*-test was employed to evaluate the statistical significance of the differences between the two groups. For experimental designs comprising multiple groups, statistical significance was assessed using one-way analysis of variance, with Duncan’s method employed for mean separation. For statistical evaluation, GraphPad Prism software (version 8; GraphPad Software, San Diego, CA, USA) served as the primary analytical platform, facilitating all data computations and graphical representations. The probability value (*p* < 0.05) was considered significant. * and ** denote *p* < 0.05 and *p* < 0.01, respectively. For RNA-seq and ChIP-seq analyses, an FDR threshold of < 0.05 was applied. The normality of the data was verified using the Shapiro–Wilk test.

## 3. Results

### 3.1. RNA Sequencing Data Overview

**Figure 1:**
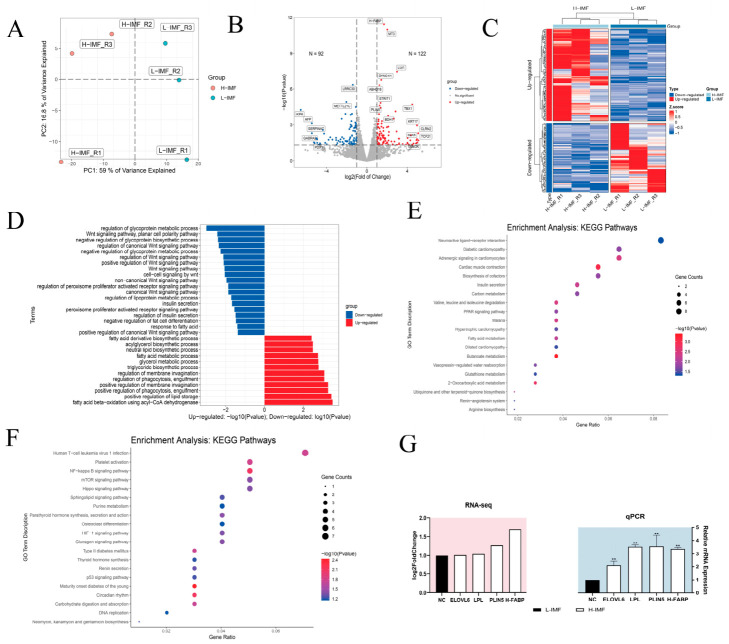
(**A**) PCA (*n* = 3). The two-dimensional cluster plot depicts the internal structure of the high and low intramuscular adipose tissue datasets in terms of variance (*n* = 3). (**B**) Volcano plot of DEGs (*n* = 3). (**C**) Heatmap of upregulated and downregulated genes in RNA-seq (*n* = 3). Z-score is used to demonstrate how many times the standard deviation (X-mean)/SD of the data has been adjusted up or down from the mean. (**D**) GO enrichment analysis of upregulated and downregulated gene sets. (**E**,**F**) KEGG pathway enrichment profiling of the transcriptomic data. (**G**) Expression changes in lipid-related genes in RNA-Seq were analyzed by qPCR (** *p* < 0.01）.

RNA-Seq was performed to characterize the transcriptomic landscape of yaks with divergent IMF phenotypes. Principal component analysis (PCA) revealed distinct clustering between H-IMF and L-IMF groups (Figure 1A). Gene expression pattern analysis revealed that compared with the L-IMF group, the H-IMF group had 122 significantly upregulated genes, including *H-FABP*, *STRIT1*, *PLIN5*, and *BDH1*, and 92 significantly downregulated genes (Figure 1B,C). Functional annotation clustering demonstrated that the DEGs were predominantly associated with metabolic terms, specifically in lipid catabolism, medium-chain fatty acid processing, and the biosynthesis of fatty acids. Specifically, functional terms associated with 12 genes were predominantly upregulated, whereas those linked to 19 genes were downregulated (Figure 1D). KEGG pathway mapping indicated that upregulated DEGs were implicated in canonical lipid-associated pathways, including *PPAR* signaling, fatty acid degradation, carbon metabolism, and insulin secretion (Figure 1E). Conversely, downregulated genes were primarily involved in regulatory cascades such as NF-KB, mTOR, *HIF-1*, and glucagon signaling pathways, which modulate lipid synthesis and lipolysis (Figure 1F). RNA-seq data were validated by qPCR (Figure 1G), confirming the robustness of our transcriptomic profiling.

### 3.2. Characteristics of SEs in LD Tissues

**Figure 2:**
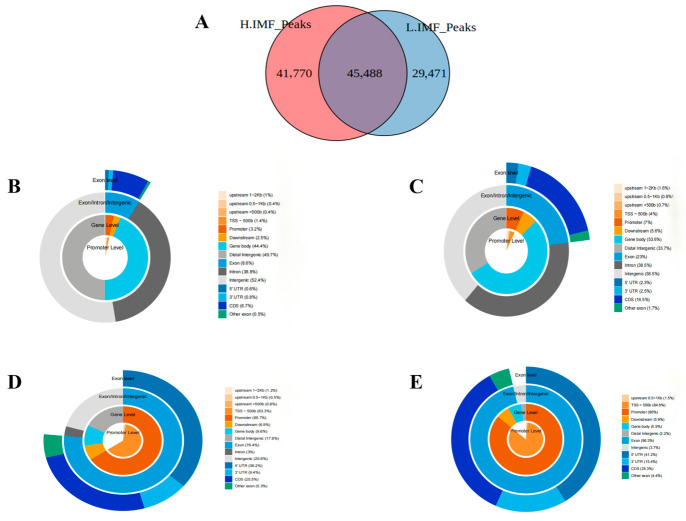
(**A**) Venn plot of DEGs in overlapping H3K27ac signal peaks in H-IMF and L-IMF. (**B**,**C**) Pie charts depicting the genomic localization of specific peaks within H-IMF and L-IMF groups. (**D**,**E**) Characterization of the distribution landscape of SEs associated with specific peaks across the genome and gene loci in H-IMF and L-IMF cohorts.

To map the enhancer repertoire associated with IMF content, we conducted H3K27ac ChIP-seq analysis. A total of 41,770 unique peaks were identified in the high-fat cohort, compared with 29,471 in the low-fat counterpart. Comparative analysis demonstrated that these datasets intersect at 45,488 loci, representing the core acetylated regions conserved across both phenotypes (Figure 2A). The genome-wide enrichment profile of H3K27ac demonstrates a distinct spatial pattern, with signal peaks distributed not solely within gene promoters but also across various gene-body-associated functional domains (Figure 2B,C). Furthermore, SEs are distributed in the promoter region, gene body region, exon, and intron regions (Figure 2D,E).

### 3.3. Identification and Functional Analysis of SE-Related Candidate Genes

**Figure 3:**
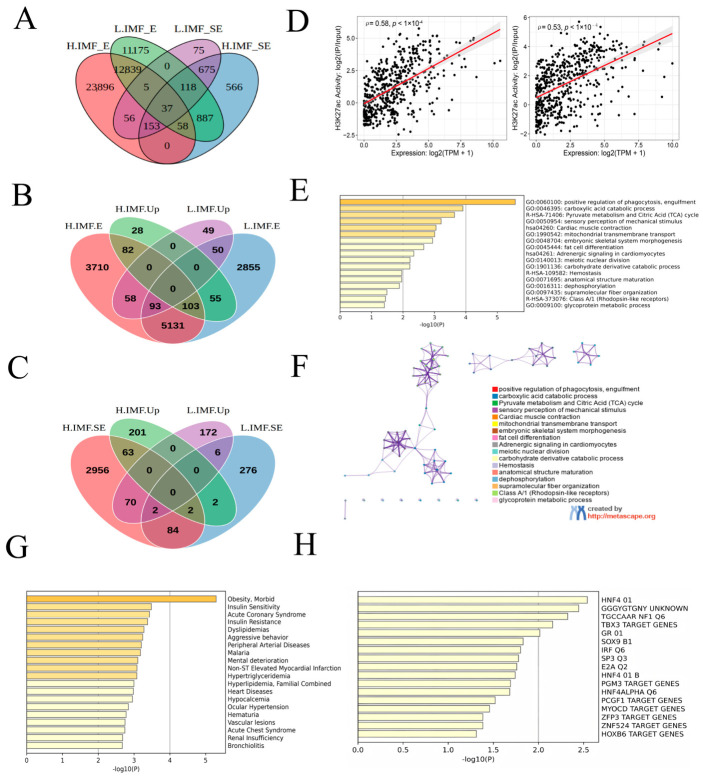
(**A**) Venn plot of enhancers and SEs in the two groups. (**B**) Venn plots of upregulated enhancer-related genes in H-IMF or L-IMF groups. (**C**) Venn plots of the upregulated SE-related genes in H-IMF and L-IMF groups. (**D**) Assessment of the association between H3K27ac signal intensity and transcriptional levels in L-IMF (right) and H-IMF (left) groups. (**E**,**F**) GO (**E**) and KEGG (**F**) enrichment analyses of 63 SE-related upregulated genes in the H-IMF group. (**G**) GO enrichment analysis of disease-associated genes upregulated in 63 SE. (**H**) GO enrichment analysis of transcription factors associated with 63 SE upregulated genes.

To investigate whether the genes with differential expression are regulated by enhancers, we conducted a comprehensive analysis of RNA-seq data and ChIP-seq data. The results revealed that 12,839 enhancers and 675 SEs were identified in H-IMF and L-IMF groups (Figure 3A). In the H-IMF group, 82 significantly upregulated genes were potentially regulated by enhancers, among which 63 were potentially regulated by SEs. Conversely, in the L-IMF group, 50 significantly upregulated genes were potentially regulated by enhancers, among which 6 were potentially regulated by SEs (Figure 3B,C). A strong positive correlation was observed between H3K27ac enrichment and transcript abundance across the genome (Figure 3D). Furthermore, functional annotation through GO and KEGG pathway enrichment of the 63 upregulated SE-associated genes identified in the H-IMF group indicated significant enrichment in processes including carboxylic acid catabolic pathways, adipogenic differentiation, glycoprotein metabolic regulation, and the pyruvate–TCA cycle axis, highlighting their potential roles in metabolic and adipose-related functions (Figure 3E,F). In addition, these genes have been implicated in diseases such as “insulin resistance”, “dyslipidemia behavior”, “peripheral arterial disease”, and “hypertriglyceridemia” (Figure 3G). 17 transcription factors were obtained that regulate these genes, such as “IRF Q6”, “SP3 Q3”, “E2A Q2”,” HNF4 01 B”, and “PGM3 target gene” (Figure 3H).

### 3.4. BDH1 Knockdown Suppresses the Proliferation and Differentiation of YIMAs

**Figure 4:**
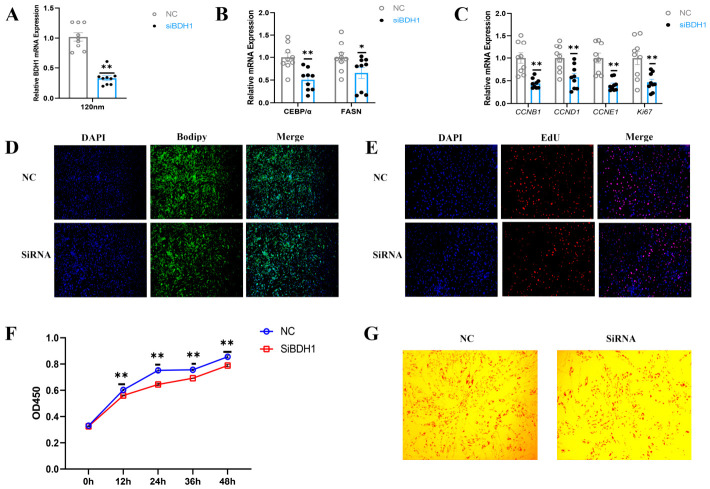
Inhibition of *BDH1* inhibits lipid deposition in YIMAs. (**A**) mRNA expression of *BDH1* at 48 h after transfection with *BDH1*-siRNA (*n* = 9, 3 biological replicates × 3 technical replicates). (**B**,**C**) Attenuation of the adipogenic (**B**) and proliferative (**C**) signature following *BDH1* silencing (*n* = 9, 3 biological replicates × 3 technical replicates). (**D**) Cellular lipid droplet staining using Bodipy. (**E**) EdU staining. (**F**) CCK-8 assay (*n* = 6, 3 biological replicates × 2 technical replicates). (**G**) Oil Red O staining. Values are presented as mean ± SEM. Statistical significance is indicated as * *p* < 0.05 and ** *p* < 0.01.

Functional characterization of the SE-associated gene *BDH1* in YIMAs was performed through RNA interference-mediated gene silencing using *BDH1*-targeting siRNA, aiming to assess its regulatory role in cellular proliferation and adipogenic differentiation. Transfection with siRNA resulted in a robust > 65% reduction in *BDH1* mRNA expression (** *p* < 0.01, Figure 4A). Consequently, the protein levels of the differentiation markers CCAAT/enhancer-binding protein alpha (*C/EBPα*) and fatty acid synthase (*FASN*) were concomitantly downregulated (*p* < 0.01, Figure 4B). Notably, *BDH1* knockdown also exerted a significant inhibitory effect on the proliferative signature, as evidenced by the reduced expression of Cyclin B1 (*CCNB1*), Cyclin D1 (*CCND1*), Cyclin E1 (*CCNE1*) and MKI67 (*Ki67*) (** *p* < 0.01, Figure 4C). Moreover, *BDH1* inhibition significantly decreased the lipid droplets content using BODIPY detection and Oil Red O staining (Figure 4D,G). The functional validation revealed that the EdU-positive cells in the interference group were significantly reduced (Figure 4E), and the CCK-8 assay confirmed that the cell viability also significantly decreased (** *p* < 0.01, Figure 4F). These results indicated that the inhibition by *BDH1* regulates proliferation and differentiation markers, thereby inhibiting the proliferation and adipogenesis of YIMAs.

### 3.5. BDH1 Overexpression Promotes Proliferation and Differentiation of YIMAs

**Figure 5:**
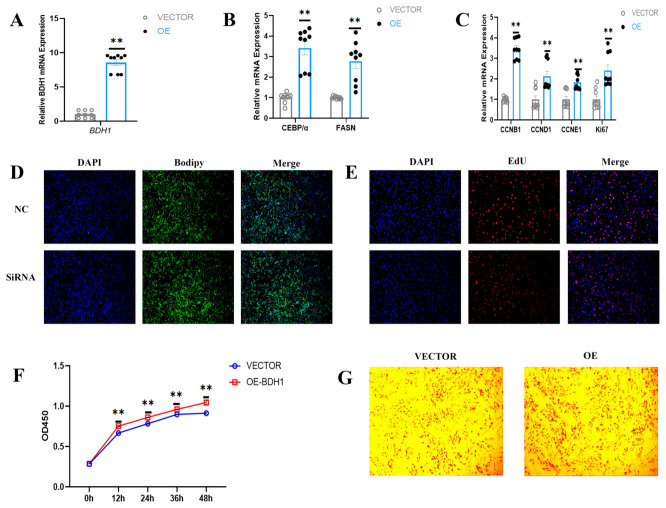
*BDH1* overexpression drives the accumulation of IMF in yak. (**A**) Transfection efficiency of pcDNA3.1-*BDH1* vector (*n* = 9, 3 biological replicates × 3 technical replicates). (**B**,**C**) Impact of *BDH1* overexpression on the mRNA levels of marker genes related to adipocyte differentiation (**B**) and proliferation (**C**) (*n* = 9, 3 biological replicates × 3 technical replicates). (**D**) Cellular lipid droplet staining using Bodipy. (**E**) EdU staining. (**F**) CCK-8 assay (*n* = 6, 3 biological replicates × 2 technical replicates). (**G**) Oil Red O staining. Values are presented as mean ± SEM. Statistical significance is indicated as ** *p* < 0.01.

To substantiate the functional role of *BDH1* during YIMA proliferation and differentiation, gain-of-function models were established via transfection with *BDH1*-overexpressing plasmids. RT-qPCR analysis confirmed a significant upregulation of *BDH1* transcript levels in pcDNA3.1-*BDH1*-transfected YIMAs compared to control cells (** *p* < 0.01, Figure 5A). To further characterize the adipogenic potential, we assessed the expression of key differentiation markers. Our data demonstrated that *BDH1* overexpression robustly increased *C/EBPα* and *FASN* mRNA expression (** *p* < 0.01, Figure 5B). Through staining experiments, we found that the number of lipid droplets increased significantly (Figure 5C,D). Consistent with these findings, a significant induction of key cell cycle regulators *CCNB1*, *CCND1*, *CCNE1*, and *Ki67* was observed at the transcriptional level (** *p* < 0.01, Figure 5C), suggesting a molecular basis for enhanced proliferation. This was further substantiated by functional assays: EdU incorporation and CCK-8 assays revealed a higher proportion of proliferating cells in the *BDH1*-overexpressing group compared to controls (Figure 5E), along with increased cell growth and viability (Figure 5F). Collectively, we conclude that *BDH1* positively regulates lipid deposition and preadipocyte proliferation in YIMAs.

### 3.6. Enhancer Activity of SE-BDH1 Genomic Components

**Figure 6:**
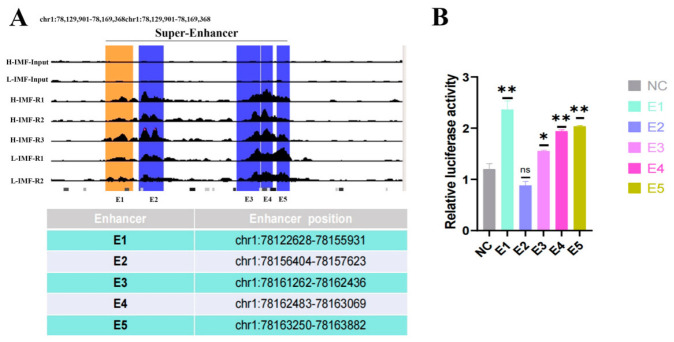
(**A**) Transcriptional activity of SE-*BDH1* sub-regions. (**A**) H3K27ac ChIP-seq profile at the *BDH1* locus across tissues with divergent expression levels. The *y*-axis represents normalized read density (reads per million), and the *x*-axis denotes linear genomic coordinates. (**B**) Validation of *BDH1* enhancer activity (E1–E5) using dual-luciferase reporter assays (*n* = 6, 3 biological replicates × 2 technical replicates). Data are presented as mean ± SEM; statistical comparisons: * *p* < 0.05; ** *p* < 0.01; ns = not significant. Data are derived from three biologically independent replicates.

Multiple component enhancers often collectively form an SE. To gain further mechanistic insights into the SE regulating *BDH1*, we partitioned it into five distinct constituent enhancer regions (E1–E5) guided by the H3K27ac enrichment profiles, ensuring each segment encompassed a specific H3K27ac peak (Figure 6A). Individual enhancer elements were subcloned into the pGL3-Promoter vector to determine their regulatory capacity. Dual-luciferase assays demonstrated that E1, E4, and E5 exhibited robust transcriptional activity, significantly outperforming the negative control (** *p* < 0.01, Figure 6B), whereas E2 demonstrated no significant activation. Collective evidence supports a functional linkage between *BDH1* and SE elements, implicating SE-mediated regulatory control in the expression dynamics of *BDH1*.

## 4. Discussion

Yak is an important endemic genetic resource in China, living in the extreme survival environment of high-altitude, low-oxygen plateaus [27]. Although yak meat is rich in nutrients, its low IMF content affects its flavor, tenderness, and texture, thereby posing challenges for meat processing and marketing [27,28], hindering the sales and economic growth of yak products, and limiting the economic benefits of yak meat production on the Qinghai–Tibet Plateau [3]. Deep-coverage whole-genome sequencing has revealed the pivotal role of SEs as master regulators for cell identity. They are located near key genes and non-coding RNAs (ncRNAs) and regulate cellular biological processes [29]. Existing studies suggest that SEs have significant associations with physiological and pathological processes [30,31,32,33]. Therefore, the systematic identification of SE-associated genes governing IMF deposition in yaks offers a robust framework for precision breeding, thereby improving meat quality and bolstering the economic sustainability of yak husbandry.

IMF is a crucial indicator for evaluating meat quality traits, such as shear force and water loss. Primarily distributed in the gaps between muscle fibers, IMF loosens connective tissue and reduces the physical hardness of muscle fiber bundles, thereby decreasing shear force and improving meat tenderness [34]. Research has shown that for every 1% increase in IMF content in yak meat, the sensory juiciness score rises notably. During chewing, the lipids released from the fat provide a lubricating effect in the mouth, which helps relieve the dryness often associated with lean meat [35]. Furthermore, IMF serves as an important carrier for flavor precursors. Yak meat with high IMF is rich in unsaturated fatty acids and free amino acids. These compounds promote the generation of characteristic volatile flavors—such as hexanal, nonanal, and 1-octen-3-ol—imparting a rich meaty and fatty aroma. In contrast, yak meat with low IMF lacks these flavor compounds, resulting in a blander overall taste and a slight gamey off-flavor [36]. IMF content also influences the processing properties of yak meat, such as water retention after curing. During the curing process, moderately deposited intramuscular fat can prevent the excessive denaturation of myofibrillar proteins. This reduces the loss of free water during curing and significantly improves the water-holding capacity of the meat [35].

To investigate the regulatory mechanisms, a multiomics dataset was constructed by leveraging ChIP-seq and RNA-seq technologies on LD samples stratified based on IMF deposition levels in yaks. This strategy facilitated the identification of 63 SE-related genes exhibiting upregulated expression in high-IMF tissues, with *BDH1* identified as a key candidate within this enriched subset. Initially isolated from rat liver and human heart tissues in 1992, *BDH1* has since been characterized across a phylogenetically diverse range of species [37,38]. Accumulating evidence from bovine, caprine, and bubaline models underscores its pivotal role as a central regulator of lipid metabolism in mammals [39,40]. Previous studies have demonstrated that the functional interplay between *BDH1* and *SREBP1* represses lipid homeostasis within buffalo mammary epithelial cells [28]. Ni et al. reported that *BDH1* acts as a negative regulatory factor in lipid metabolism in Goat mammary epithelial cells [40]. Ashley, S. et al. found that in a type 2 diabetes Apoe^−^/^−^ mouse model, elevated *BDH1* expression drives lipid catabolism and reduces lipid accumulation through enhanced ketone body production, thereby suppressing aortic plaque development and attenuating diabetes-associated atherosclerosis [16]. Based on previous findings, we investigated the regulatory role of *BDH1* during IMF deposition in yaks. Functional characterization (including overexpression and interference experiments) revealed that key lipid metabolism markers were significantly affected. RT-qPCR results revealed that *BDH1* overexpression promotes proliferation and differentiation, while knockout inhibits proliferation and differentiation. Hence, as an SE-associated gene, *BDH1* influences lipid metabolism in yak IMF deposition through positive regulation.

SEs, as the primary regulators of cell identity genes, are closely related to the transcriptional regulation of fat metabolism and related characteristics. The analysis of SEs in chicken adipose tissue revealed their tissue-specific regional control function for fat, providing crucial insights into the upstream mechanisms of genes involved in fat deposition [41]. *BDH1,* identified in the present study as a key gene involved in energy metabolism, has a potential regulatory role in IMF deposition in yaks, controlled by SEs. This is similar to the mechanism observed in glioma stem cells, where specific SEs regulate key pathways of fatty acid synthesis to maintain cell-specific functions, supporting the EGFR signaling pathway by driving polyunsaturated fatty acid synthesis, thereby validating the conserved regulation of metabolism-related core pathways by SEs [42]. IMF-specific deposition is based on SEs, which regulate *BDH1,* allowing yaks to adapt to the hypoxic environment of high altitudes. This is consistent with the tissue-specific and functional-specific characteristics of SE regulation in different species. For example, the SE landscape in chicken adipose tissue illustrates significant tissue specificity, and there are also species-specific molecular mechanisms in the regulation of bovine adipogenesis. In the future, combined approaches such as RNA pull-down and dual-luciferase reporter assays will help identify SE regions regulating *BDH1* and clarify whether their effect are mediated through lncRNA-mediated epigenetic mechanisms or direct interaction with transcription factors. This work significantly advances the mechanistic comprehension of adipogenesis by deciphering the regulatory landscape underlying IMF deposition in yaks.

The function of *BDH1* goes far beyond its fundamental role in regulating lipid metabolism and intramuscular fat (IMF) deposition. It also possesses important theoretical significance and great application potential for the quality improvement and industrial utilization of yak meat. From the perspective of genetic breeding, *BDH1* can be regarded as a promising candidate gene and molecular marker for meat quality trait selection, and can be incorporated into molecular marker-assisted breeding systems. Studies have found that the *BDH1* gene plays a crucial regulatory role in ketone body metabolism within the skeletal muscle, liver, and heart tissues of pigs. The expression level of *BDH1* is significantly higher in Chinese indigenous pig breeds compared to exotic breeds. Furthermore, a specific upstream insertion mutation may enhance the gene’s expression, thereby influencing the proliferation and differentiation of muscle cells, and ultimately altering the growth performance and meat quality of pigs [34]. Polymorphisms of the *BDH1* gene are significantly correlated with key economic traits of yaks, including IMF content, marbling score and muscle tenderness. Genotype screening technology based on the *BDH1* gene enables the early selection of superior individuals. This approach effectively overcomes the limitations of traditional phenotype-based selection methods, such as long breeding cycles and significant interference from environmental factors, thereby providing a reliable molecular target for the genetic improvement of yak germplasm resources.

In future research, targeted nutritional regulation strategies, such as altering dietary energy levels or supplementing functional probiotics and prebiotics, can be applied to remodel rumen microbial composition [43]. Rumen microbial modulation can further mediate the expression of *BDH1* in rumen epithelium and skeletal muscle tissues. Such nutritional interventions not only improve the high-altitude adaptability of yaks, but also promote IMF deposition via optimizing energy metabolism and nutrient partitioning, thereby realizing the directional and standardized production of high-quality yak meat for industrial application.

Although we identified the regulatory effects of SE-associated genes on IMF content, this study still has several limitations. (1) There are numerous different yak breeds, and the Maiwa yaks used in this study may not be representative of all yaks, as yaks from different regions exhibit variations. (2) The inaccessibility of pure yak IMF necessitated the use of H-IMF and L-IMF muscle tissues as surrogate samples, potentially constraining the precision of our data interpretation. (3) The relatively small sample size (*n* = 3 per group) used in the initial RNA-seq analysis is a limitation regarding the broad statistical applicability of this study. Nevertheless, this approach is frequently adopted in discovery-driven omics research on specialized livestock. To compensate for this, we conducted strict validation of the key genes in a larger sample set during subsequent experiments. (4) Although our integrative analysis indicates an association between SEs and BDH1 expression, the direct causal relationship and three-dimensional chromatin interactions await further experimental validation in future studies. Furthermore, the absence of genetic intervention strategies precluded rigorous functional interrogation of proposed regulatory models. Notwithstanding these constraints, our identification of the SE-associated gene *BDH1* suggests a putative pivotal role in IMF deposition, the regulatory intricacies of which in lipid metabolism remain to be fully elucidated.

## 5. Conclusions

In this study, RNA-Seq and H3K27ac antibody-mediated ChIP-seq analysis was conducted using LD muscle tissues with high and low IMF content from yaks. In the H-IMF group, 82 enhancer-related upregulated genes were identified, among which 63 were key SE-related regulating IMF deposition. Among these candidate genes, we demonstrated that *BDH1* is subject to SE-mediated regulation and positively regulates IMF deposition, which was further confirmed through functional assays.

## Data Availability

The original contributions presented in this study are included in the article/Supplementary Materials. Further inquiries can be directed to the corresponding author.

## Supplementary Materials

The following supporting information can be downloaded at: https://www.mdpi.com/article/10.3390/foods15101811/s1, Table S1. Primers utilized in this study; Table S2. Gene interference sequence in this article; Table S3. GroupID; Table S4. Overlapping genes between SE-related genes and significantly upregulated DEGs in the H-IMF group.
